# Overestimation of the classification model for Raman spectroscopy data of biological samples

**DOI:** 10.1128/msystems.00063-25

**Published:** 2025-03-10

**Authors:** Ivan A. Bratchenko, Lyudmila A. Bratchenko

**Affiliations:** 1 Laser and Biotechnical Systems Department, Samara National Research University, Samara, Russia; Katholieke Universiteit Leuven, Leuven, Belgium

**Keywords:** surface-enhanced Raman spectroscopy, classification model, overestimation, vaginal fluid, liquid biopsy

## LETTER

In recent years, Raman spectroscopy and surface-enhanced Raman spectroscopy (SERS) have demonstrated progress in disease detection and classification and are widely used in optical and liquid biopsy (1–7). The combination of Raman spectroscopy and machine learning techniques offers a way for precise analysis of spectral data and extremely accurate detection of different diseases (8). However, the complex nature of Raman spectral data and the difficulties in modern machine learning algorithm implementations often lead to an overoptimistic result (8). In this regard, it is necessary not only to collect Raman data during liquid or optical biopsy but also to create a reliable model of spectral data analysis.

In their recent publication, Wen et al. (9) demonstrated the combination of SERS and machine learning techniques for the detection and classification of bacterial vaginosis during the spectral analysis of human vaginal fluid. The authors demonstrated more than 99% accuracy; however, the presented results may be treated incorrectly due to the overestimation of the proposed classification models.

The authors of the commented paper utilized different machine learning algorithms (CNN, convolutional neural network; SVM, support vector machine; DT, decision tree; XGBoost; AdaBoost; RF, random forest; GBoost) and reasonably divided the spectral data into training and test sets. However, the main text of the commented paper presented accuracy for the training set only (Table 2 in the commented paper), and Wen et al. discussed this value in the abstract and discussion of the commented paper. However, test set (or validation set) accuracy is presented in the supplemental file of the commented paper (Table S2), and accuracy values for validation and training differ dramatically.

For convenience, we collected values of training and validation accuracies in Table 1. As one may see, there is a dramatic difference in validation and training accuracies for all presented classifiers. The least difference of 13% is observed for CNN, whereas for RF and GBoost the difference is more than 45%. Such huge changes between training and test accuracies prove that the proposed models are overestimated (8, 10–12).

**TABLE 1:** Comparison of different machine learning algorithms for the training and independent validation (test) sets

Algorithm	Accuracy of validation	Accuracy of training
CNN	87.20%	99.97%
SVM	81.75%	97.58%
DT	79.70%	92.92%
XGBoost	67.45%	95.83%
AdaBoost	61.35%	93.27%
RF	52.10%	96.71%
GBoost	46.10%	94.46%

One possible reason for the model’s overestimation is the collection of multiple spectra from one sample. During classification model construction and validation (Fig. S2 in the commented paper), there is a possibility to utilize spectra from one sample both in training and validation (11). In such a scenario, the classification model is already familiar with the validation data and will demonstrate an excellent result. Next, in the test with the independent validation data, such a model provides lower accuracy as it deals with unknown data, and only the value of accuracy obtained for the unknown test data may be utilized as the true accuracy value for the proposed classifier.

In general, the commented paper provides novel insights into the possibilities of SERS combined with machine learning techniques to diagnose bacterial vaginosis during the spectral analysis of human vaginal fluid. However, the stated accuracy of bacterial vaginosis in the abstract (99%) is overoptimistic and should be replaced with a lower accuracy of 87% obtained for the independent validation set.
